# Validity and Reliability of the Six-Minute Walking Test Compared to Cardiopulmonary Exercise Test in Individuals with Heart Failure Systematic Review and Meta-Analysis

**DOI:** 10.3390/jcm14238303

**Published:** 2025-11-22

**Authors:** Garyfallia Pepera, Varsamo Antoniou, Eleni Karagianni, Ladislav Batalik, Jing Jing Su

**Affiliations:** 1 Clinical Exercise Physiology and Rehabilitation Research Laboratory, Department of Physiotherapy, Faculty of Health Sciences, University of Thessaly, 35132 Lamia, Greece; gpepera@uth.gr (G.P.); varsamoantoniou@uth.gr (V.A.); ekaragiann@uth.gr (E.K.); 2 Department of Rehabilitation, University Hospital Brno, 62500 Brno, Czech Republic; 3 Department of Physiotherapy and Rehabilitation, Faculty of Medicine, Masaryk University, 62500 Brno, Czech Republic; 4 Rehabilitation Clinic, Faculty of Medicine, Masaryk University, 62500 Brno, Czech Republic; 5 Department of Public Health, Faculty of Medicine, Masaryk University, 62500 Brno, Czech Republic; 6 School of Nursing, The Hong Kong Polytechnic University, Hong Kong; jjsu@twc.edu.hk

**Keywords:** heart failure, cardiopulmonary exercise testing, six-minute walk test, functional assessment, validity, reliability

## Abstract

**Background:** Reduced cardiorespiratory fitness along with poor exercise tolerance are regarded as potential morbidity and mortality predictors within the heart failure (HF) population. Despite the reliability and accuracy of the gold-standard cardiopulmonary exercise test (CPET) for assessing cardiorespiratory fitness, its complexity and tolerability issues among HF patients mean that the 6 min walk test (6MWT) is a cost-saving and well-tolerated complementary assessment. We aimed to systematically review the validity, reliability, and safety of the 6MWT compared to CPET for patients with HF. **Methods:** This study is a systematic review and meta-analysis. Embase, Medline, and Scopus were searched from inception to November 2023. We applied Fisher’s z-transformation to correlation coefficients and pooled effects under a random-effects model; heterogeneity (I^2^), leave-one-out sensitivity, and Egger’s test were reported. **Results:** Twenty studies were finally included, involving 5379 HF participants. A significant moderately strong positive correlation was shown between the 6MWT distance and CPET peak oxygen consumption: (r) = 0.62, 95% CI 0.58–0.66; I^2^ = 56.95%; *p* < 0.001. The results showed an excellent test–retest reliability, with a pooled intraclass correlation coefficient of 0.93 (95% CI 0.89–0.95; I^2^ = 92.06%; *p* < 0.001). A pooled weighted mean difference of 15.5 m (95% CI 10.2–20.8) was found for the learning effect between the first and second 6MWT. Although some patients required rest stops or reported symptoms such as fatigue or dyspnea, no 6MWTs were terminated due to serious adverse events. **Conclusions:** Compared with CPET, the 6MWT distance demonstrated a moderately strong correlation with peak VO_2_, excellent test–retest reliability, and a small learning effect. The 6MWT can therefore complement CPET or serve as a pragmatic alternative when CPET is not feasible; it does not replace comprehensive CPET assessment.

## 1. Introduction

Heart failure (HF) is a major cause of death and hospitalization worldwide. According to the European Society for Cardiology guidelines, the prevalence of HF in the general population ranges from 2% to 3%, increasing with age and reaching 10–20% in those aged 70 to 80 years [1]. Patients’ quality of life can be significantly impacted, since they often report symptoms of reduced functional ability, poor exercise tolerance, and shortness of breath during physical activity [2].

Maximal tests aiming at the assessment of the cardiorespiratory fitness of HF patients are often complex and costly, and patients’ progression of HF stages makes their application debatable [3,4]. Maximal aerobic capacity is inversely correlated with the severity of HF and directly correlated with prognosis and life expectancy [5]. The direct cardiorespiratory assessment of peak oxygen consumption (VO_2_peak, sometimes referred to as VO_2_max) in the maximal symptom-limited cardiopulmonary exercise test (CPET) is considered the gold-standard measure of exercise capacity. However, patients with HF may find it difficult to cooperate with the test due to its complexity or their severe functional impairment [6]. As a complementary test, the 6MWT is a low-cost, easily reproducible test that reflects physical exertion levels associated with daily activities [7]. In the original 6MWT protocol, participants are instructed to walk on a 30 m course repeatedly and cover as much distance as possible within six minutes [8]. Research has demonstrated that the 6MWT is responsive to treatment interventions, can predict morbidity and mortality, and may provide a more accurate measure of a patient’s ability to perform daily activities compared to other exercise-based performance tests [9]. Several high-quality references support the claim that the 6MWT is a valid, reliable, and sensitive assessment tool for evaluating functional capacity in the HF patient population [10].

Although both CPET and the 6MWT are widely used for HF patients, prior reviews have not jointly quantified the convergent validity of 6MWD with CPET-derived peak VO_2_, the test–retest reliability (including the learning effect), and the safety profile of the 6MWT specifically in HF patients. Our systematic review and meta-analysis address this gap to clarify when 6MWT can complement CPET and when it can act as a practical alternative.

## 2. Materials and Methods

### 2.1. Design

A comprehensive search was conducted in MEDLINE (via PubMed; from 1946), Embase (from 1947), and Scopus (from database inception) through 30 November 2023, as pre-specified in the PROSPERO registration (CRD42023494697). Search strategies followed PRISMA 2020 guidance [11].

### 2.2. Eligibility Criteria

The eligibility criteria for this study were guided by the PICOS framework. The inclusion criteria were the following: (1) population: individuals with a medical diagnosis of HF; (2) intervention: participation in a well-defined 6MWT assessment; (3) comparison: CPET or standard care reference; (4) outcomes: measures of reliability, validity, or adverse events; and (5) study design: clinical trials, prospective, and retrospective cohort studies that described repeated 6MWTs, or compared 6MWD with established reference tests (CPET).

Studies not in the English language, conference abstracts, case studies, and studies without available full texts were excluded.

### 2.3. Search Methods

A comprehensive literature search was conducted across three electronic databases. The PICOS framework guided the search strategy. Additionally, a hand search of bibliographies from relevant review articles was performed to identify further studies. Search strings (MeSH/keywords) are provided in Supplementary Materials (including ‘heart failure’, ‘six-minute walk test’, ‘validity’, ‘reliability’, ‘correlation’, ‘peak oxygen uptake’, ‘cardiopulmonary exercise test’).

### 2.4. Study Selection

Two reviewers (LB and GP) independently conducted the initial screening of studies based on titles and abstracts, progressing to full-text reviews. Disagreements about study inclusion were resolved through discussion with a third researcher (JJS). The inter-rater agreement (Cohen’s κ) for the screening process was calculated and reported.

### 2.5. Study Quality and Risk of Bias

We assessed methodological quality using a pre-specified checklist adapted from Bellet et al. [12] with binary (yes/no) items, applied independently by two reviewers with consensus adjudication; κ values are reported. A brief mapping of our Bellet-adapted quality checklist to COSMIN domains, including per-study domain ratings and overall risk-of-bias judgments, is provided in Supplementary Table S2.

### 2.6. Data Extraction

All study data were extracted independently by two authors (LB and GP), and any disagreement about interpretation was resolved by a third author (JJS). The authors developed a table for data extraction, which included the following: (a) origin of the articles, including authors, year, and country; (b) sample characteristics, such as sample size, setting, age, and diagnosis; (c) group design, including a brief description of the assessment protocol; (d) assessment results; (e) correlation coefficient; and (f) intraclass correlation coefficient (ICC).

### 2.7. Data Analysis and Synthesis

Correlation coefficients were Fisher z-transformed and pooled using a random-effects model (MedCalc), with results back-transformed to r. Heterogeneity was summarized with I^2^, and leave-one-out sensitivity analyses were performed. Small-study effects were explored with Egger’s regression. For reliability, we pooled ICCs (primary analysis) and summarized the Pearson r narratively when ICCs were unavailable. The learning effect was synthesized as a paired mean difference (T2–T1).

Strength of linear relationship: At least 0.8 is very strong; 0.6 up to 0.79 is moderately strong; 0.3 to 0.59 is fair; less than 0.3 is poor [13].

## 3. Results

Figure S1 presents the PRISMA flow diagram illustrating the search results. Initially, 1408 articles were identified across the three databases. Following the removal of 1065 duplicates, 343 studies remained for relevance assessment. After screening titles and abstracts, the full texts of 67 studies were examined for eligibility. Ultimately, there are a total of 20 representative articles in this review [6,8,14,15,16,17,18,19,20,21,22,23,24,25,26,27,28,29,30,31].

### 3.1. Study Characteristics

The studies were conducted across 11 countries in Asia, Europe, and America, including a total of 5379 participants (Table 1). The sample sizes varied from 15 to 2054 individuals. Participants had a mean age of 49 to 69 years, with a majority being male (76.3%). Recruitment was primarily conducted in clinical settings, notably cardiac units and outpatient clinics, with some participants also being sourced from research centers. The diagnoses covered in the studies included the HF New York Heart Association Functional Classification (NYHA) classification grades I–IV [32,33], mostly grades II–III. The major HF etiologies were dilated cardiomyopathy (48.3%) and ischemic heart disease (42.1%). The mean left ventricular ejection fraction was 29.1%.

### 3.2. Risk of Bias

In Table S1, the quality assessment of the articles included in this review is presented. Several studies underwent assessment in multiple categories, such as reliability and validity. The methodological quality of these studies differed based on the specific appraisal evaluation relevant to each study type. Notably, one study [25] (11%) in terms of reliability and five studies [6,25,28,30,34] (29%) for validity demonstrated a high quality. Additionally, 65% and 67% of studies exhibited medium quality in relation to validity and reliability, respectively.

### 3.3. Reliability

Out of the 20 studies examined, 9 studies [14,15,17,22,23,25,26,27] demonstrated a strong test–retest reliability (intraclass correlation coefficient ranging from 0.82 to 0.98) between repeated 6MWDs. An insignificant difference was found for repeated tests between 6MWD-T1 and 6MWD-T2 [15,17,21], and between 6MWD-T2 and 6MWD-T3 [17]. A significant difference between 6MWD-T1 and 6MWD-T2 was reported in one study [21]. These differences were 1% to 7% between 6MWD-T1 and 6MWD-T2 [8,21], and 1% between 6MWD-T2 and 6MWD-T3 [17]. A learning effect was reported between 5m and 31m [15,22,26]. A minimal clinically important difference between 6MWD-T1 and 6MWD-T2 was found for 35.2 m and 39.4 m, respectively [22,23].

### 3.4. Validity

**Figure 1:**
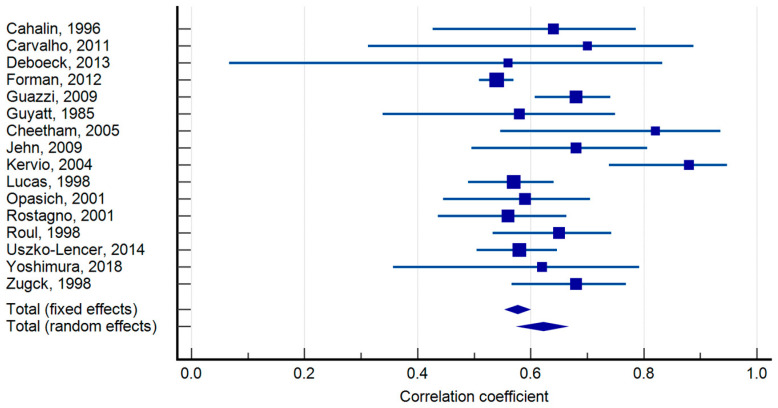
Forest plot showing the correlation coefficients between 6MWD and CPET peak VO2 [6,8,14,15,16,18,19,20,21,22,24,25,27,28,29,30].

**Figure 2:**
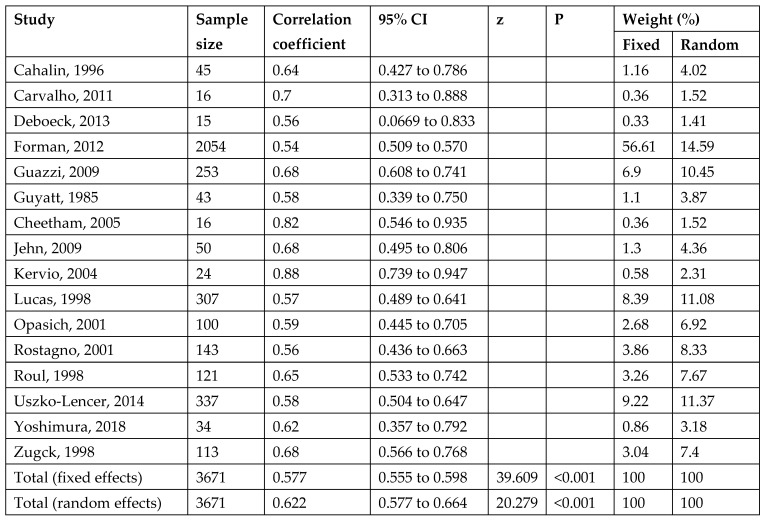
Results of pooled correlation coefficients between 6MWD and CPET peak VO2 [6,8,14,15,16,18,19,20,21,22,24,25,27,28,29,30].

**Figure 3:**
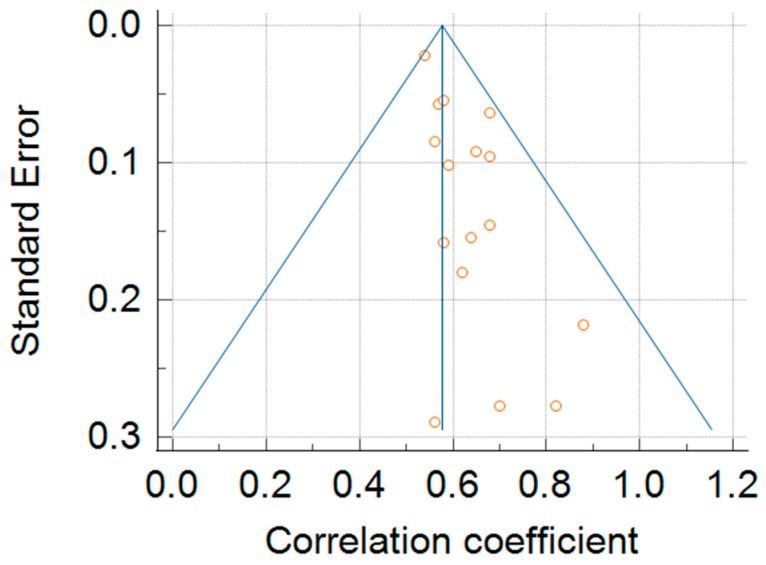
Funnel plot for publication bias analysis.

Between-study heterogeneity for the 6MWD–peak VO_2_ correlation was moderate (I^2^ = 56.95%). A leave-one-out analysis reduced heterogeneity to ~I^2^ ≈ 41%, while maintaining a similar pooled correlation, indicating the robustness of the association (Figure 1, Figure 2). For test–retest reliability, the heterogeneity was high (I^2^ = 92.06%); nevertheless, all study-level ICCs were high (0.82–0.98), supporting a consistently excellent reliability across protocols and settings (Figure S2). The funnel plot analysis suggested small-study effects (Egger’s *p* < 0.01) (Figure 3).

Most (17/20) studies compared the 6MWT with the CPET as a reference test, the most common of which were measurements taken during symptom-limited CPET on bicycle [6,8,14,15,16,22,24,25,27,28,29,30,34] or treadmill ergometers [18,19,21]. Pearson’s correlation coefficient was reported in many of these studies; Table 1 shows the details of the comparisons performed.

The correlation of the 6MWD with peak oxygen uptake during symptom-limited CPET was moderate to high (r = 0.4 to 0.88, *p* < 0.05) in 16 articles. Other studies examined the correlation of the peak heart rate in 6MWT with the peak heart rate in symptom-limited CPET; two medium-quality studies reported a fair to moderately strong correlation (r = 0.51 and 0.66, *p* < 0.05) [15,25]. Moreover, Zugck et al. reported that the 6MWT peak heart rate was 70% (SD 20%) of the peak heart rate during symptom-limited CPET [25]. One high-quality study [29] and one moderate-quality study [24] reported a fair but significant correlation between ventilatory threshold symptom-limited CPETs and the 6MWD (r = 0.54 and 0.54). Two very strong correlations were founded in medium-quality studies [22,25] between the maximal watts during symptom-limited CPET and 6MWDs (r = 0.68, r = 0.69 *p* < 0.05). A medium-quality study [15] reported a moderate correlation (r = 0.57, *p* < 0.05) between the peak oxygen uptake during symptom-limited CPET and the peak oxygen uptake during 6MWT. One strong correlation between 6MWD and NYHA classification was found (r = −0.65, *p* < 0.05), and one fair correlation (r = 0.42, *p* < 0.05) was reported between the maximal systolic blood pressure during symptom-limited CPET and 6MWT [25,34].

### 3.5. Adverse Events and Symptoms Occurrence During 6MWT

A total of 9/20 studies (in 968 HF patients) providing 6MWTs reported the occurrence of adverse events or complications during assessment [6,8,14,15,21,25,26,27,28]. Four studies providing 6MWTs reported that assessments were performed safely without adverse events, and none of the assessments were prematurely terminated [14,21,25,26]; two studies reported that the assessments were safe [8,15]. Roul et al. reported that 4/121 (3%) patients required a rest and stop during 6MWT and 34/121 (28%) reported symptoms (fatigue or dyspnea) during the test [27]. The symptoms were mild in 16/34 patients and moderate to severe in 18/34, but no 6MWTs were prematurely terminated. The study by Guazzi et al. similarly reported that 8/253 (3%) patients required a rest stop, and 25% complained of symptoms (fatigue and dyspnea) during the test [6].

### 3.6. Meta-Analysis of Test–Retest Reliability of 6MWT

**Figure 4:**
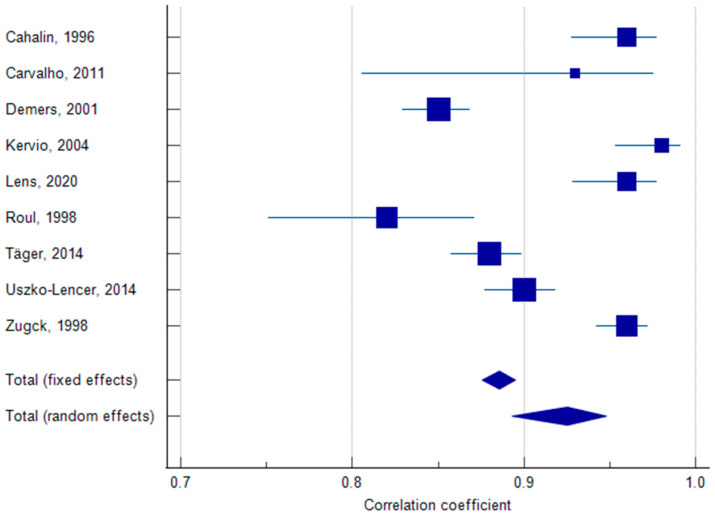
Forest plot showing the correlation coefficients between 6MWD-T1 and 6MWD-T2 [14,15,17,21,22,23,25,26,27].

**Figure 5:**
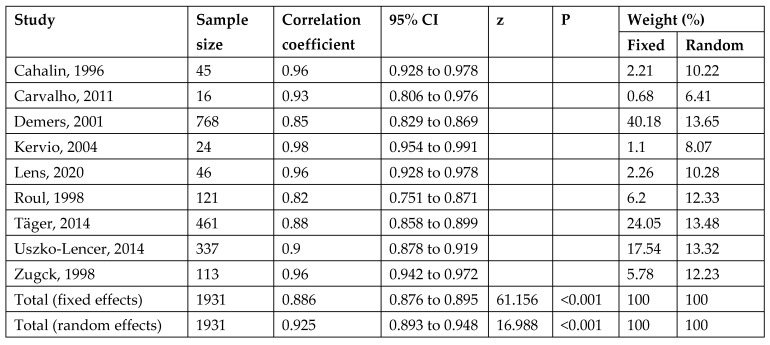
Meta-analysis of pooled correlation coefficients between 6MWD-T1 and 6MWD-T2 [14,15,17,21,22,23,25,26,27].

A meta-analysis of ICC values between the 6MWD-T1 and 6MWD-T2 reported by nine studies, including 1931 participants, was pooled. The results showed a significant correlation [ICC = 0.93, 95% confidence interval (0.89, 0.95), I^2^ = 92.06%, *p* < 0.001] (Figure 4 and Figure 5). The high heterogeneity was not resolved by a sensitivity analysis; however, the included studies had ICCs ranging from 0.82 to 0.98, indicating strong positive correlations across studies.

## 4. Discussion

This review integrates three decision-critical dimensions of the 6MWT in HF patients—the convergent validity versus CPET peak VO_2_, test–retest reliability with an explicit quantification of the learning effect, and safety—to inform a pragmatic assessment when CPET is not feasible [35]. We found a moderately strong association between 6MWD and peak VO_2_, excellent reliability (ICC ≈ 0.93), a small learning effect (~15 m), and no serious adverse events reported, suggesting that the 6MWT can complement CPET in clinical decision-making [36]. Importantly, the 6MWT does not replace comprehensive CPET indices (e.g., VE/VCO_2_ slope, ventilatory reserve).

More specifically, the meta-analysis results of this current systematic review assessing the validity of the 6MWT towards the CPET, regarding the peak VO_2_ uptake, revealed a moderate to high positive correlation. Similar positive correlations emerged in a previous study [37], thus further enhancing the assumption that a 6MWT may act as an adjunct to CPET for the evaluation of the HF patients’ cardiorespiratory fitness. It needs to be mentioned that parameters such as the peak VO_2_ and the VO2 max are proposed as the best indicators of the functional capacity in patients with HF [6]. Therefore, integrating easy-to-implement, low-cost, and validated assessment tools such as the 6MWT into the evaluation procedures for patients with HF is of crucial importance. 

Moreover, according to the recent guidelines, the most appropriate way to prescribe and perform safe and adequate physical activity for the HF population is via the use of the peak HR obtained in a symptom-limited stress test [38]. In this current systematic review, the comparison of the peak HR achieved in the CPET and 6MWT led to the observation of a fair to moderately strong correlation (r = 0.51 and 0.66, *p* < 0.05) [15,25]. Moreover, Zugck et al. reported that the 6MWT peak heart rate was 70% (SD 20%) of the peak heart rate during symptom-limited CPET [25]. The aforementioned results are in accordance with another study that reported a moderate but significant correlation between the 6MWT and CPET peak HR (r = 0.66, *p* = 0.005) [15]. Therefore, the quantification of exercise intolerance and the prescription of exercise for HF patients could be implemented via the use of a 6MWT when a CPET is not feasible to be conducted.

The observed heterogeneity likely reflects CPET modality (cycle vs. treadmill), HF phenotypes, 6MWT protocol differences (track length, ATS/ERS protocol adherence), and timing between repeated tests. Given this, the pooled estimates should be interpreted as average effects across diverse settings rather than context-invariant constants; nonetheless, the direction and clinical meaning remained stable in sensitivity analyses. The evidence of small-study effects suggests that summary correlations may be overestimated; we interpret the results with an appropriate caution.

Taking into account the correlation between the functional status assessed by the NYHA classification and the 6MWD, one included study proposed a strong correlation (r = −0.65, *p* < 0.05) [20]. Notably enough, previous studies [39,40] and systematic reviews [41] have revealed a mild-to-moderate inverse correlation between the functional status assessed by the NYHA classification and the 6MWD. The HF participants in the Jehn et al. study were of NYHA I and II [20]. Bearing in mind that the NYHA classification performs better in more symptomatic patients (NYHA III/IV) [41], the strong correlation between the NYHA and 6MWD revealed in this systematic review could be explained. This relationship demonstrates how the subjective assessment of functional capacity through the NYHA classification aligns with the objective measure of exercise tolerance provided by the 6MWT. Moreover, improvements in the NYHA functional class have been associated with an increased 6MWD, indicating that enhancements in symptoms and functional status are reflected in an improved exercise capacity [42]. Additionally, both the NYHA classification and 6MWD have been associated with patient-reported health status and quality of life in heart failure patients [43]. The NYHA class is indicative of functional capacity, with higher classes reflecting more severe symptoms and limitations, which are mirrored in a reduced 6MWD [44]. This relationship underlines the value of utilizing both the NYHA classification and the 6MWT in evaluating the clinical status and prognosis of heart failure patients.

Furthermore, a learning effect has been found; thus, two assessments are proposed at the initial assessment to ensure the accuracy of the 6MWT. This learning effect is consistent with similar studies among patients with asthma [45] or other chronic respiratory diseases, as well as healthy individuals [46,47]. The learning effect established in the current study indicates that many HF patients could have an underestimation of their functional capacity if only one 6MWT was performed. This is supported by the magnitude of the learning effect of the 6MWT in this study, which suggests within-patient variability for repeated 6MWTs. Thus, the learning effect on the second 6MWT is demonstrated, to our knowledge, for the first time in a large sample of HF patients, and the findings suggest the need for performing two 6MWTs in clinical settings and studies.

Additionally, in the included study of Uszko et al. addressing a sample size of 337 HF patients, the learning effect appeared to be less significant in older individuals with a severe respiratory deficiency and severe HF [22]. Similarly, another study reported a lack of a learning effect in HF populations with poor baseline functions and a severely impaired functional capacity [48]. This absence of the learning effect can probably be explained by the hypothesis that HF frail individuals are performing each 6MWT close to their maximum exercise capacity; thus, the possibility for a between-6MWT difference is minimized.

The recent literature further underscores the pragmatic value of the 6MWT alongside CPET in HF patients. For instance, a 2024 study and meta-analysis in cardiac populations (including HF patients with preserved ejection fraction) reported a moderate to good screening accuracy of the 6MWT for identifying severely reduced functional capacity against CPET VO_2_ thresholds, supporting our validity findings [49]. Moreover, a 2025 meta-analysis synthesized VO_2_peak and 6MWD disparities between HF patients with a reduced ejection fraction and HF patients with a preserved ejection fraction, reinforcing phenotype-specific considerations when interpreting 6MWT performance [50].

The use of the 6MWT as a potential, easy-to-implement endpoint tool for the assessment and the quantification of the overall response to interventions for the HF population is a factor that needs to be addressed [51]. Similarly, other field-based submaximal tests, such as the six-minute step test, have also demonstrated a good reliability and validity in patients with HF [52], supporting the broader applicability of simple functional assessments in this population. In patients with HF, the existing literature [53,54] suggests a minimal clinically important difference (MCID) ranging from 22 to 90 m. In line with this, the included studies of the present review reported MCID values between 35 and 39 m [22,23]. It is worth mentioning that it is proposed that an MCID as low as ~36 m over a 6–12-month period [23] or 14 m at 12 weeks of intervention [55] can be used as a threshold for the follow-up evaluation of the efficiency of the interventions aimed at the improvement of the HF patient’s exercise capacity.

The safety of exercise testing is of crucial importance. It requires attention to avoid any adverse events or relapses in the HF patient’s health status [56]. The results of this systematic review proclaim the safety of the 6MWT, since in most of the included studies an absence of any adverse events was reported. Even in studies where safety issues were reported, the symptoms did not result in premature termination of the 6MWT procedure [6,8,14,15,21,25,26,27,28]. Similar results, proposing the safety of 6MWT, are noted in several studies [57,58].

The recent COVID-19 pandemic has prompted the development of new approaches for assessing the functional capacity of HF patients when face-to-face evaluation is not feasible [59]. Based on these results, this systematic review supports the potential of tele-accelerometry via the 6MWT as an easier-to-implement alternative for evaluating the functional status of HF patients [34].. The results from a previous study in patients with diabetes mellitus reinforce the validity and reliability of the tele-assessed, outdoor 6MWT to adequately assess functional capacity [60]. Finally, a recent study demonstrated that the automated treadmill 6MWT appears to be an acceptable tool with adequate validity, reliability, and responsiveness for assessing functional capacity in patients utilizing cardiac rehabilitation programs [61].

### Limitations

Despite conducting a thorough search of databases, relevant articles were overlooked. Secondly, the adapted quality assessment tool was pragmatic and not COSMIN-based, potentially leading to misclassifying the study quality; we acknowledge this and provide a brief COSMIN mapping in the Supplementary Materials. Thirdly, a significant limitation is the frequent use of cycle CPET (~77%) as the reference; walking and cycling performance may differ due to modality specificity. The included sample is predominantly male (76%) and consists mainly of HFrEF in NYHA II–III; therefore, the generalizability to women, HFpEF, and the very elderly is limited. The search was pre-specified in PROSPERO to end on 30 November 2023; very recent publications may not have been included. The reporting of CPET–6MWT intervals and disease duration was inconsistent and is noted as a limitation. We contextualized our findings with the recent literature, while keeping pre-specified pooled outcomes unchanged; a living review update under PRISMA 2020 could be considered.

## 5. Conclusions

Overall, compared with CPET, the 6MWT shows a clinically meaningful convergent validity for functional capacity, an excellent reliability with a small learning effect, and a favorable safety profile. It can complement CPET or serve as a practical alternative when CPET is unavailable, while recognizing that it cannot replace the CPET-derived ventilatory metric.

## Figures and Tables

**Table 1:** Characteristics of studies.

Reference	NYHA	Diagnosis (%)	LVEF (%)	Sex (*n*)	Age	6MWT Protocol	6MWT Mean	CPET Protocol	mL/kg/min	Correlations	Reliability 6MWT
Cahalin (1996) [14] USA	3.3 (0.6)	DCM 73, Ischemic 22 Myocarditis 5	20.0	45 Men 40 Women 5	49.0 (8)	Guyatt 1985 [8]	310 (100)	Bicycle ergometer (Ramp)	12.2 (4)	r = 0.64 * DW-6MWT/pVO2-CPET	0.96
Carvalho (2011) [15] Brazil	I–II	DCM 29 Other 71	31.4	16 Men 12 Women 4	57.5 (10)	ATS	550 (66)	Bicycle ergometer (Ramp)	14.1 (4)	r = 0.70 * DW-6MWT/pVO2-CPET	0.93
Deboeck et al. (2013) [16] Belgium	I–III	DCM 20 Ischemic 60 Other 20	31	15 Men 13 Women 2	52	ATS	445 (95)	Bicycle ergometer (Ramp)	13.9 (3)	r = 0.56 * DW-6MWT/pVO2-CPET	-
Demers et al. (2001) [17] Canada	I–IV	Ischemic 71 Other 29	27	768 Men 637 Women 131	63 (11)	Lipkin 1986	381 (84)	NR	NR	NR	0.85
Forman et al. (2012) [18] USA	II–IV	DCM 49 Ischemic 51	24.9	2054 Men 1459 Women 595	59	ATS	372	Treadmill (modified Naughton)	14.6	r = 0.54 * DW-6MWT/pVO2-CPET	
Guazzi et al. (2009) [6] Italy	2.2 (0.8)	DCM 39 Ischemic 61	36.3	253 Men 199 Women 54	62 (10)	Roul 1998 [27]	351	Bicycle ergometer (Ramp)	15 (4.7)	r = 0.68 * DW-6MWT/pVO2-CPET	
Guyatt et al. (1985) [8] USA	II–IV	NR	NR	43 Men 34 Women 9	65 (8)	Guyatt 1985 [27]	NR	Bicycle ergometer (Ramp)	NR	r = 0.58 * DW-6MWT/pVO2-CPET	NR
Cheetham (2005) [19] Australia	III–IV	DCM 31 Ischemic 63 Other 6	23	16 Men 14 Women 2	59 (3)	Guyatt 1985 [27]	458 (21)	Treadmill (Bruce protocol)	16.3 (1.1)	r = 0.82 * DW-6MWT/pVO2-CPET	
Jehn et al. (2009) [20] Germany	I–III	DCM 28 Ischemic 42 Other 30	39	50 Men 38 Women 12	61 (14)	NR	510 (129)	Bicycle ergometer (Ramp)	20.5	r = 0.68 * DW-6MWT/pVO2-CPET	
Kervio et al. (2004) [21] France	II–III	DCM 79 Ischemic 21	27	24 Men 19 Women 5	65	Guyatt 1985 [27]	427	Treadmill (Weber protocol)	16.7	r = 0.88 * DW-6MWT/pVO2-CPET	0.98
Lans et al. (2020) [26] Sweden	II–III	DCM 26 Ischemic 61 Other 13	29	46 Men 37 Women 9	68 (9)	ATS	411 (96)	NR	NR	NR	0.96
Lucas et al. (1998) [28] USA	NR	NR	23	307 Men 240 Women 67	52 (13)	Lipkin 1986	393 (104)	Bicycle ergometer (Ramp)	14 (5)	r = 0.57 * DW-6MWT/pVO2-CPET	-
Omar et al. (2017) [31] USA	I–IV	NR	30	433 Men 320 Women 113	56	NR	NR	NR	NR	r = 0.4 * DW-6MWT/pVO2-CPET	NR
Opasich et al. (2001) [29] Italy	II–III	DCM 40 Ischemic 41 Other 19	26	100 Men 87 Women 13	53 (9)	NR	396 (92)	Bicycle ergometer (Ramp)	14.6 (4)	r = 0.59 * DW-6MWT/pVO2-CPET	-
Rostagno et al. (2001) [30] Italy	II–III	DCM 36 Ischemic 37 Other 27	33	143 Men 78 Women 65	57	Guyatt 1985 [27]	NR	Bicycle ergometer (Ramp)	NR	r = 0.56 * DW-6MWT/pVO2-CPET	-
Roul et al. (1998) [27] France	II–III	DCM 47 Ischemic 29 Other 24	30	111 Men 99 Women 22	59(11)	Guyatt 1985 [27]	433 (108)	Bicycle ergometer (Ramp)	17 (4.5)	r = 0.65 * DW-6MWT/pVO2-CPET	0.82
Täger et al. (2014) [23] Germany	I–III	NR	31.5	461 Men 360 Women 101	57 (12)	NR	480 (106)	NR	NR	-	0.88
Uszko-Lencer (2017) [22] Netherlands	I–III	NR	35	337 Men 235 Women 102	65	ERS/ATS	473	Bicycle ergometer (Ramp)	15.5	r = 0.58 * DW-6MWT/pVO2-CPET	0.90
Yoshimura (2020) [24] Japan	II–III	DCM 56 Ischemic 3 Other 41	37	34 Men 13 Women 21	69	ATS	412 (10)	Bicycle ergometer (Ramp)	15.8 (4)	r = 0.62 * DW-6MWT/pVO2-CPET	-
Zugck et al. (2000) [25] Germany	2.2 (0.8)	DCM 100	19	113 Men 90 Women 23	54 (12)	Guyatt 1985 [27]	466 (107)	Bicycle ergometer (Ramp)	15.4 (5.4)	r = 0.68 * DW-6MWT/pVO2-CPET	0.96

Dilated cardiomyopathy (DCM), left ventricular ejection fraction (LVEF), cardiopulmonary exercise test (CPET), * = significant, DW—distance walked by body weight.

## Data Availability

The original contributions presented in this study are included in the article/Supplementary Materials. Further inquiries can be directed to the corresponding author.

## Supplementary Materials

The following supporting information can be downloaded at https://www.mdpi.com/article/10.3390/jcm14238303/s1. Figure S1: Flow diagram detailing the search strategy. Figure S2: Sensitivity analysis by removing one study [21], with high ICC of 0.88, resolving the heterogeneity [6,8,14,15,16,18,19,20,22,24,25,27,28,29,30]. Table S1: Critical evaluation of included studies for reliability and validity [6,8,14,15,16,17,18,19,20,21,22,23,24,25,26,27,28,29,30,31]. Table S2: Mapping of Bellet-style checklist items to COSMIN domains with domain-level ratings and overall risk-of-bias judgments for each study [6,8,14,15,16,17,18,19,20,21,22,23,24,25,26,27,28,29,30,31].

## Abbreviations

The following abbreviations are used in this manuscript:

CPET	Cardiopulmonary Exercise Test
6MWT	Six-Minute Walk Test
HF	Heart Failure
NYHA	New York Heart Association
MCID	Minimal Clinically Important Difference
VO_2_peak	Peak Oxygen Uptake
PICOS	Population, Intervention, Comparison, Outcomes, Study Design
PRISMA	Preferred Reporting Items for Systematic Reviews and Meta-Analyses
