# Supplementary material for: Validity and Reliability of the Six-Minute Walking Test Compared to Cardiopulmonary Exercise Test in Individuals with Heart Failure Systematic Review and Meta-Analysis
Source: J Clin Med. 2025 Nov 22;14(23):8303. doi: 10.3390/jcm14238303 (PMC12693657; doi:10.3390/jcm14238303)
Supplement: Supplementary file 1 [file jcm-14-08303-s001.zip › jcm-3949170-supplementary-Figures and Table S1.pdf]

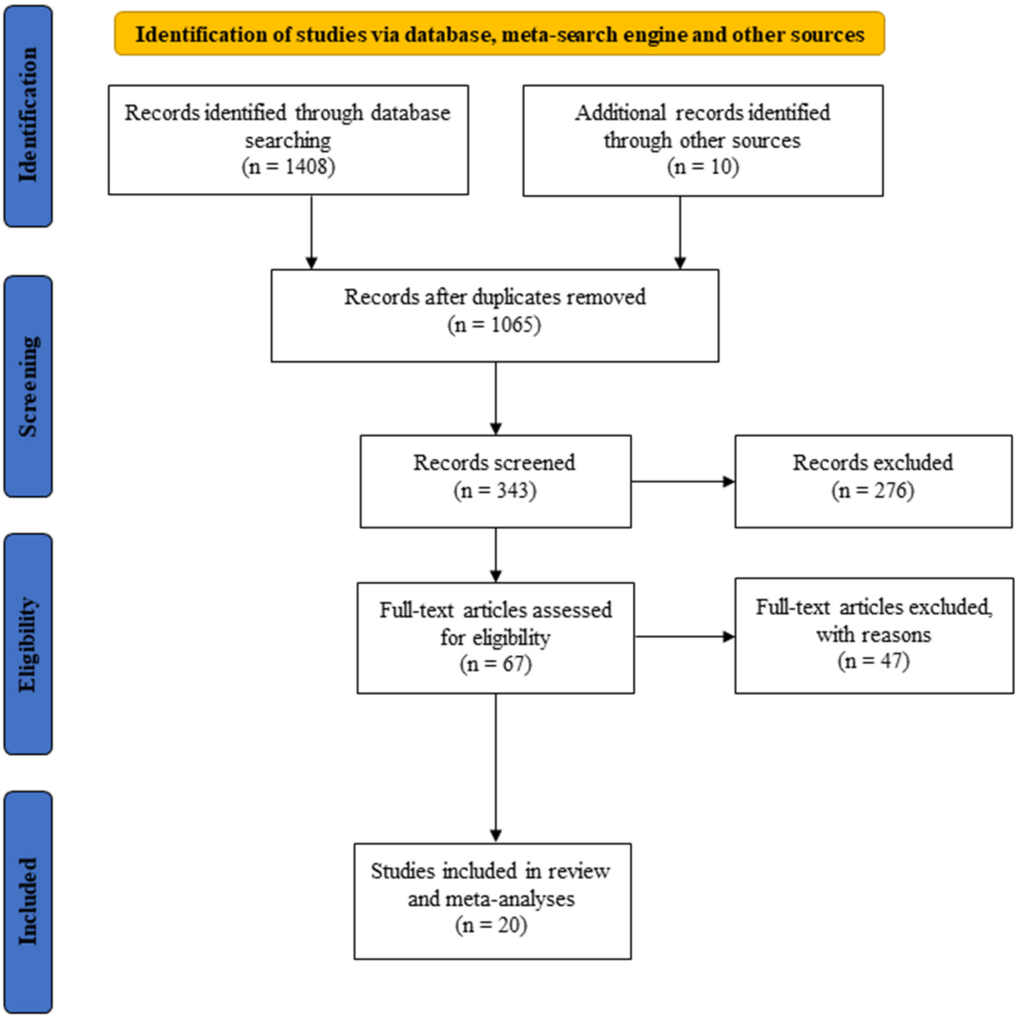

Figure S1 Flow diagram detailing the search strategy.

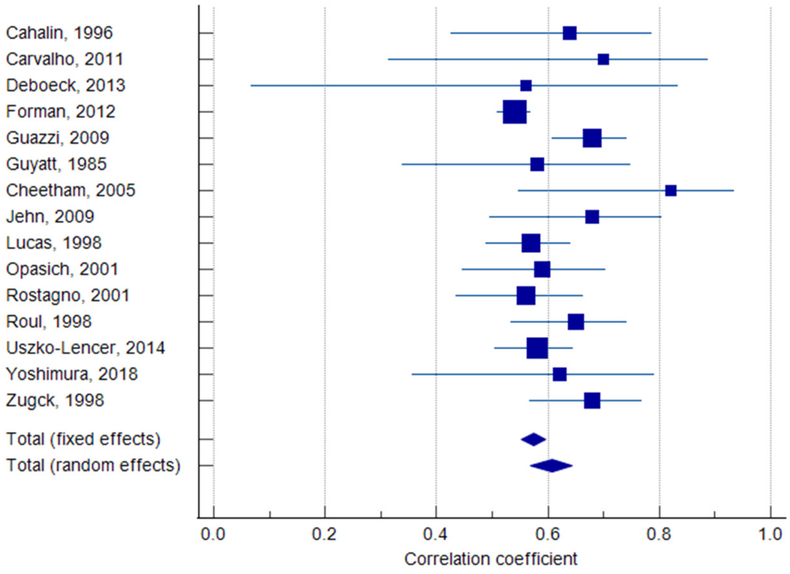

Study	Sample size	Correlation coefficient	95% CI	z	P	Weight (%)	
						Fixed	Random
Cahalin, 1996	45	0.640	0.427 to 0.786			1.17	3.28
Carvalho, 2011	16	0.700	0.313 to 0.888			0.36	1.14
Deboeck, 2013	15	0.560	0.0669 to 0.833			0.33	1.05
Forman, 2012	2054	0.540	0.509 to 0.570			56.94	19.44
Guazzi, 2009	253	0.680	0.608 to 0.741			6.94	11.17
Guyatt, 1985	43	0.580	0.339 to 0.750			1.11	3.15
Cheetham, 2005	16	0.820	0.546 to 0.935			0.36	1.14
Jehn, 2009	50	0.680	0.495 to 0.806			1.30	3.61
Lucas, 1998	307	0.570	0.489 to 0.641			8.44	12.22
Opasich, 2001	100	0.590	0.445 to 0.705			2.69	6.33
Rostagno, 2001	143	0.560	0.436 to 0.663			3.89	8.09
Roul, 1998	121	0.650	0.533 to 0.742			3.28	7.24
Uszko-Lencer, 2014	337	0.580	0.504 to 0.647			9.27	12.72
Yoshimura, 2018	34	0.620	0.357 to 0.792			0.86	2.52
Zugck, 1998	113	0.680	0.566 to 0.768			3.05	6.91
Total (fixed effects)	3647	0.574	0.552 to 0.596	39.243	<0.001	100.00	100.00
Total (random effects)	3647	0.607	0.568 to 0.643	23.207	<0.001	100.00	100.00

Figure S2 Sensitivity analysis by removing one study Kervio, 2004 with high corelation coefficient of 0.88, resolved the heterogeneity

Table S1: Critical evaluation of included studies for reliability and validity.

Questions	1	2	3*	4†	5†	6*	7†	8*	9	10*	11	12	13	14	15	Yes (%)
Validity studies scored out of 12																
Cahalin, 1996	Y	Y	Y	N/A	N/A	Y	N/A	Y	Y	Y	Y	Y	N	N	N	75
Carvalho, 2011	Y	N	Y	N/A	N/A	N	N/A	Y	Y	Y	Y	N	N	N	Y	58
Deboeck, 2013	Y	N	Y	N/A	N/A	Y	N/A	Y	Y	Y	N	Y	N	N	N	58
Forman, 2012	Y	N	Y	N/A	N/A	Y	N/A	Y	Y	Y	N	Y	Y	Y	N	75
Guazzi, 2009	Y	Y	Y	N/A	N/A	Y	N/A	Y	Y	Y	Y	Y	Y	Y	Y	100
Guyatt, 1985	Y	N	N	N/A	N/A	Y	N/A	Y	Y	Y	Y	Y	Y	Y	N	75
Cheetham, 2005	Y	N	Y	N/A	N/A	Y	N/A	Y	Y	Y	N	Y	N	Y	N	67
Jehn, 2009	Y	N	N	N/A	N/A	Y	N/A	Y	Y	Y	N	Y	Y	Y	Y	83
Kervio, 2004	Y	N	Y	N/A	N/A	Y	N/A	Y	Y	Y	N	Y	N	N	Y	67
Lucas, 1998	N	N	Y	N/A	N/A	Y	N/A	Y	Y	Y	Y	Y	N	Y	Y	83
Omar, 2017	N	N	Y	N/A	N/A	N	N/A	Y	N	N	N	N	Y	Y	N	33
Opasich, 2001	N	N	N	N/A	N/A	Y	N/A	Y	Y	Y	N	Y	N	Y	N	50
Rostagno, 2001	Y	Y	Y	N/A	N/A	Y	N/A	Y	Y	Y	N	Y	Y	Y	N	83
Roul, 1998	Y	N	Y	N/A	N/A	N	N/A	Y	Y	Y	Y	Y	N	Y	N	67
Uszko-Lencer, 2014	N	N	Y	N/A	N/A	Y	N/A	Y	Y	Y	N	Y	N	Y	N	58
Yoshimura, 2018	Y	N	Y	N/A	N/A	Y	N/A	Y	Y	Y	Y	Y	N	Y	N	75
Zugck, 1998	Y	Y	Y	N/A	N/A	Y	N/A	Y	Y	Y	Y	Y	Y	Y	N	92
Reliability studies scored out of 11																
Cahalin, 1996	Y	Y	N/A	Y	N	N/A	N	N/A	Y	N/A	Y	Y	N	N	N	55
Carvalho, 2011	Y	N	N/A	N	N	N/A	N	N/A	Y	N/A	Y	N	N	N	Y	36
Demers, 2001	Y	N	N/A	N	Y	N/A	N	N/A	Y	N/A	N	Y	Y	Y	N	55
Kervio, 2004	Y	N	N/A	Y	Y	N/A	Y	N/A	Y	N/A	N	Y	N	N	Y	64
Lens, 2020	Y	Y	N/A	N	Y	N/A	Y	N/A	Y	N/A	Y	Y	Y	N	N	73
Roul, 1998	Y	N	N/A	N	N	N/A	Y	N/A	Y	N/A	Y	Y	N	Y	N	55
Täger, 2014	N	N	N/A	Y	Y	N/A	N	N/A	Y	N/A	N	Y	N	Y	N	46
Uszko-Lencer, 2014	N	N	N/A	N	N	N/A	Y	N/A	Y	N/A	N	Y	N	Y	N	36
Zugck, 1998	Y	Y	N/A	Y	Y	N/A	Y	N/A	Y	N/A	Y	Y	Y	Y	N	91

Questions (adapted from Bellet et al. [12]): (1) If human subjects were used, did the authors give a detailed description of the sample of subjects used to perform the (index) test? (2) Did the authors clarify the qualification or competence of the rater(s) who performed the (index) test? (3) Was the reference standard explained? (4) Were raters blinded to the findings of other raters? (5) Was the order of examination varied? (6) If human subjects were used, was the time period between the reference standard and the index test short enough to be reasonably sure that the target condition did not change between the two tests? (7) Was the stability (or theoretical stability) of the

variable being measured taken into account when determining the suitability of the time interval between repeated measures? (8) Was the reference standard independent of the index test? (9) Was the execution of the (index) test described in sufficient detail to permit replication of the test? (10) Was the execution of the reference standard described in sufficient detail to permit its replication? (11) Were events/withdrawals from the study explained? (12) Were the statistical methods appropriate for the purpose of the study? (13) Were participants selected either randomly or consecutively? (14) Was the number of participants either >50 or was a sample size calculation provided? (15) Did participants give consent prior to testing?

N, no; Y, yes; n/a, criteria not applicable for the study type. Questions without a superscript letter were relevant to all study types.

** Questions relevant to validity.*

† Questions relevant to reliability.
